# Sensing and identification of carbon monoxide using carbon films fabricated by methane arc discharge decomposition technique

**DOI:** 10.1186/1556-276X-9-402

**Published:** 2014-08-19

**Authors:** Elnaz Akbari, Zolkafle Buntat, Aria Enzevaee, Mahsa Khoshkhooy Yazdi, Mahdi Bahadoran, Ali Nikoukar

**Affiliations:** 1 Centre for Artificial Intelligence and Robotics (CAIRO), Universiti Teknologi Malaysia, Kuala Lumpur 54100, Malaysia; 2 Institute of High Voltage & High Current, Faculty of Electrical Engineering, Universiti Teknologi Malaysia, Johor Bahru 81310, Malaysia; 3 Faculty of Mechanical Engineering, Universiti Teknologi Malaysia, Johor Bahru 81310, Malaysia; 4 Department of Chemistry, Faculty of Science, Universiti Teknologi Malaysia, Johor Bahru 81310, Malaysia; 5 Institute of Advanced Photonics Science, Nanotechnology Research Alliance, Universiti Teknologi Malaysia, Johor Bahru 81310, Malaysia; 6 Faculty of Computing, Universiti Teknologi Malaysia (UTM), Johor Bahru 81310, Malaysia

**Keywords:** Carbonaceous materials, Gas sensing, Methane arc discharge decomposition

## Abstract

Carbonaceous materials have recently received attention in electronic applications and measurement systems. In this work, we demonstrate the electrical behavior of carbon films fabricated by methane arc discharge decomposition technique. The current-voltage (I-V) characteristics of carbon films are investigated in the presence and absence of gas. The experiment reveals that the current passing through the carbon films increases when the concentration of CO_2_ gas is increased from 200 to 800 ppm. This phenomenon which is a result of conductance changes can be employed in sensing applications such as gas sensors.

## Background

Continuous emission of carbon dioxide (CO_2_) and other greenhouse gases by industrial activities has been increased recently and has led to global warming. This calls for the need to develop low-cost, sensitive, resettable sensors that can be used to monitor the CO_2_ concentration in industrial exhaust gases [1-3].

Over the past few years, graphene and carbon nanotubes have become the center of attention in the sensor manufacturing technology [4-8]. Furthermore, their unique electrical properties such as tunable conductance and high charge mobility make them ideal for application as sensing medium in nanotechnology [9,10]. In this paper, we have designed and developed a method for the fabrication of a carbon film material implementing high-voltage AC arc discharge [11-14]. In the proposed system, pure methane in atmospheric pressure is passed over the electrodes inside a Pyrex glass tube chamber where the carbon film fabrication process takes place [15-17]. Once the arc ignites between the graphite electrodes, the methane gas starts to decompose to its constituent species. At the end of this process, a fine soot of carbonaceous material remains between the two electrodes. The material produced this way is then checked through high-resolution optical microscopy as well as scan electron microscopy (SEM) to observe the material physical and structural characteristics. Once the carbon films are grown, the measurement process is carried out.

### Arc discharge decomposition

Generally, when a voltage is applied to two electrodes, an electrical potential is created which tends to move electrons from the positive pole to the negative. This is what causes an electric flow of electrons or electric current through a wire or resistance. When there are no conductive wires and/or resistors connecting the two electrodes, i.e., there is either an insulating barrier or simply the ambient air between them, no flow of electrons occurs under normal circumstances for low voltages. In case of high-voltage arc discharge, when the voltage is increased, the methane between the electrodes is ionized. In this situation, the non-conductive medium breaks down and becomes conductive, allowing for the charge carriers to travel through it. This phenomenon occurs very fast and is usually accompanied by sparks and light emissions. As a matter of fact, the electrons inside the gap are accelerated with the applied voltage and cause electron impact ionization. When methane is present in the gap between the electrodes, it will be defragmented into carbon and hydrocarbon species. This electric arc discharge under flowing methane is then used in the experiment for carbon decomposition.

### Experimental setup

**Figure 1:**
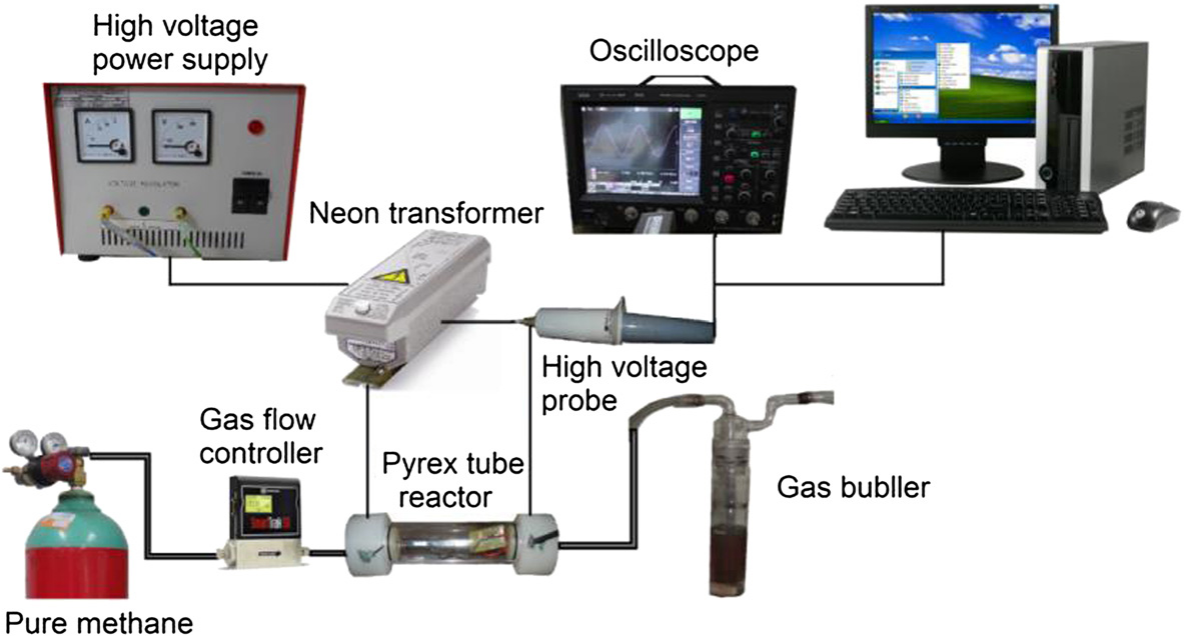
Setup of arc discharge decomposition process.

In Figure 1, the complete experimental setup for carbon film fabrication has been demonstrated.

To start the decomposition process, an insulated reactor chamber was designed and fabricated employing a Pyrex glass tube which was enclosed with two Teflon flanges at two ends to prevent gas leakage. A PCB board on which the electrodes were mounted in specific fixed distances was put in this chamber; the distance between them is 1,531 μm. One end of the Pyrex tube reactor was attached to a gas flow controller (PC-controlled, model Sierra Co. CA, USA) and the gas cylinder, while the other end was connected to a gas bubbler tube so as to absorb the pollutant gases from the reactor outlet released after the decomposition process. Different values of pure methane gas (200 to 800 ppm) were passed through the chamber using a gas flow meter. A pressure regulator was implemented to make sure the gas flow had the atmospheric pressure. Single-phase AC electrical power was fed to a high-voltage power supply with built-in amplifier to control and manipulate the operating voltage. This voltage was then increased to kilovolt scale using a step-up neon transformer. The neon transformer was used at normal operating frequency (50 Hz) to produce high voltage. This high voltage was applied to the two electrodes to start the methane decomposition process. To monitor the applied high voltage, a high-voltage probe Tektronix (20 kV DC/40 kV AC, US Patent 015-049, ×1:1,000; Beaverton, OR, USA) was coupled to a four-channel oscilloscope (LeCroy, Chestnut Ridge, NY, USA; WJ354A WaveJet 354A, 500 MHz, 4 CHs, 500 kp/Ch, 1 GS/s) to record the signal measurements. Details of the operating parameters of the arc discharge methane decomposition process are provided in Table 1.

**Table 1:** Operating parameters of carbon strands

**Parameter**	**Value**
Temperature	At room environment
Frequency	50 Hz
High voltage	1 to 26 kV
Flow rate	200 to 800 ppm
Precursor gas	Pure methane (99.99%)
Pressure	Atmospheric

### Diagnostics of the carbon film

**Figure 2:**
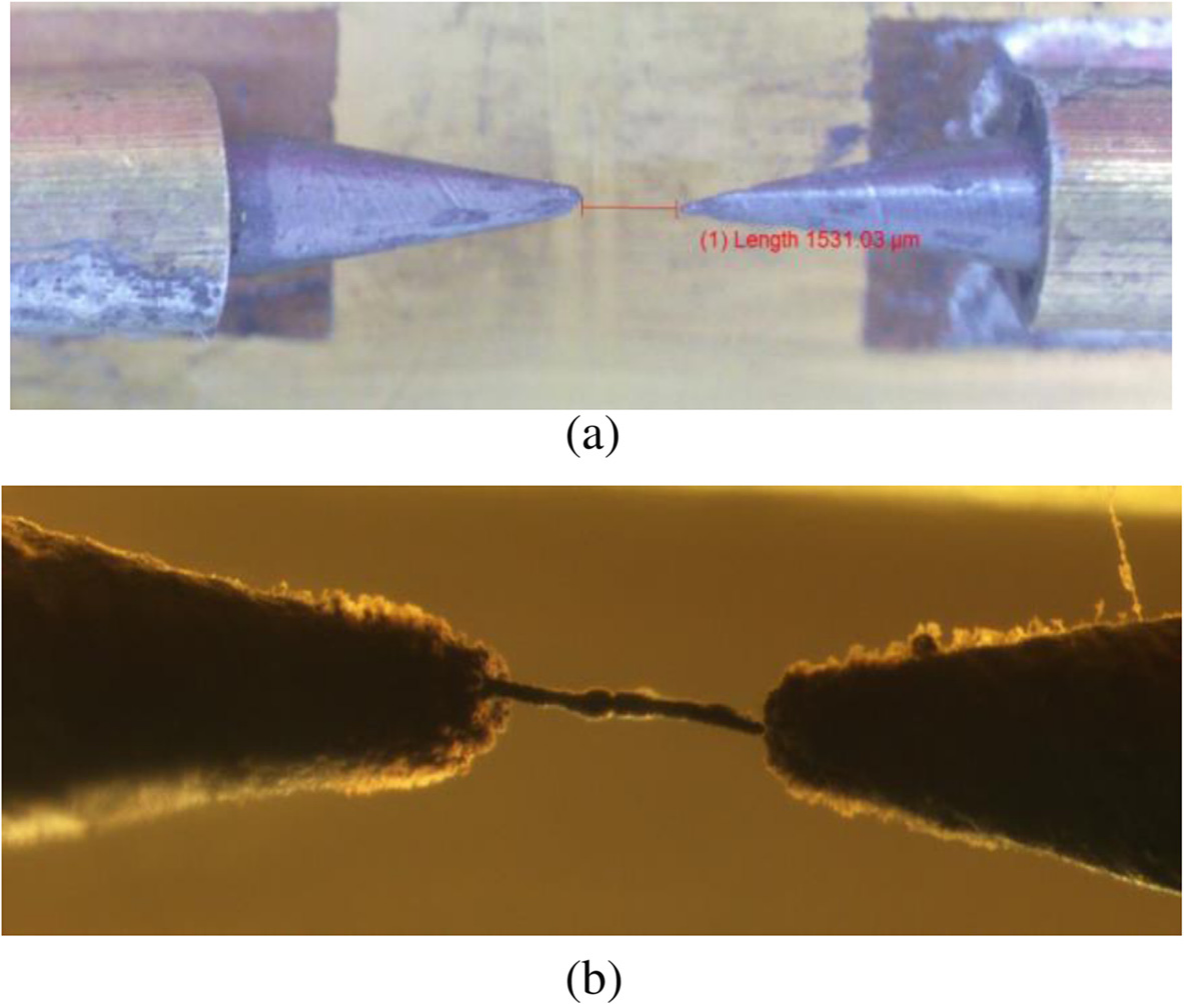
TTT electrode configuration (a) before arc discharge decomposition, (b) carbon film obtained.

Once the arc discharge is initiated, methane decomposition starts causing the resultant carbon atoms to deposit and stack up between the two electrodes creating a conductive bridge. The growth time was measured to be 11.6 s at the voltage of 16.4 kV. The carbon film fabricated in this process is inspected using high-resolution optical microscopy, as shown in Figure 2. There are three configurations for installing the electrodes on the PCB board, namely, plane to plane (PTP), tip to plane (TTP), and tip to tip (TTT); however, in this study, we have only investigated the TTT structure.

### Inspection by scan electron microscopy

**Figure 3:**
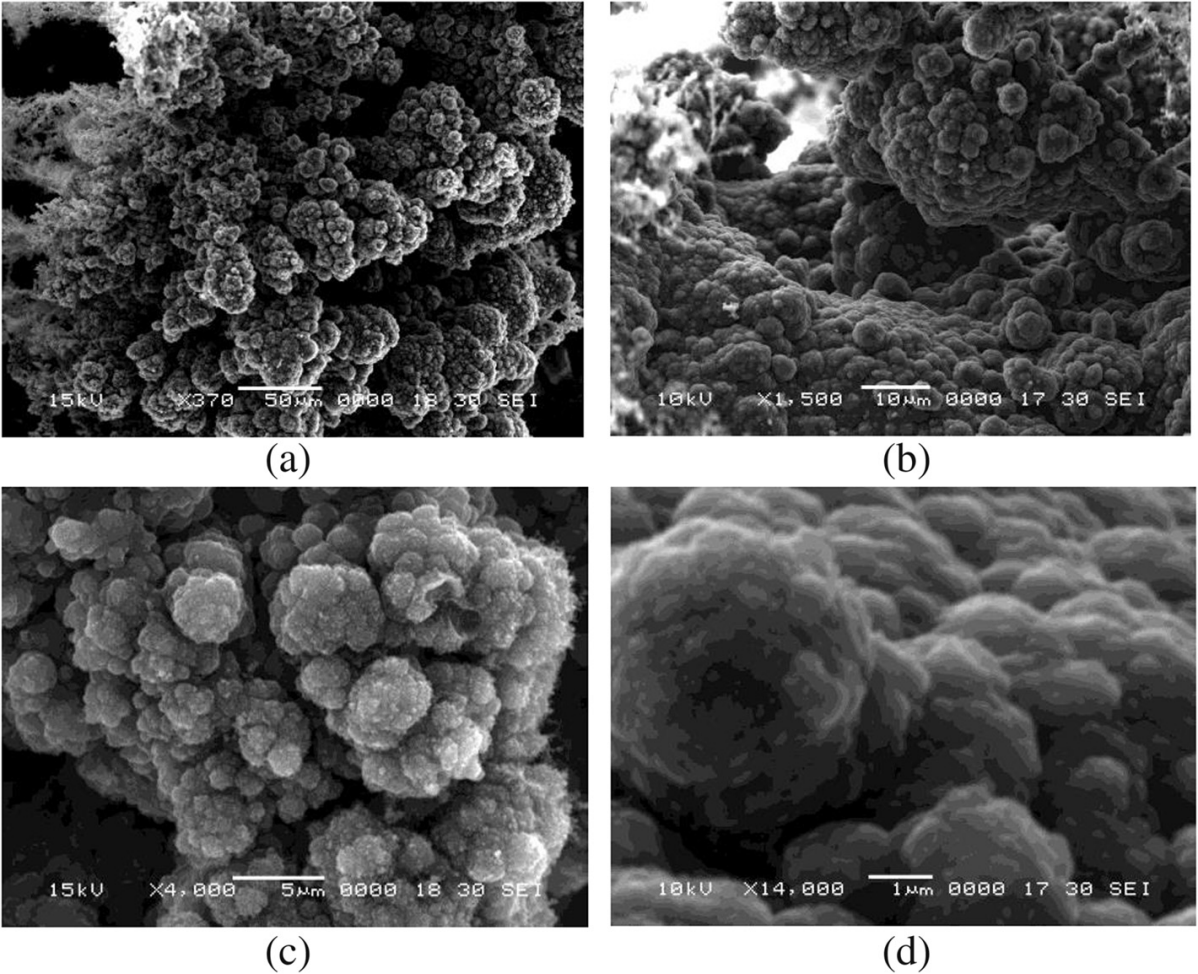
**SEM image of a sample.** Imaging mode **(a)** × 370 at 15 kV, **(b)** × 1,500 at 10 kV, **(c)** × 4,000 at 15 kV, and **(d)** × 14,000 at 10 kV.

A scanning electron microscope (SEM) scans the samples with a focused beam of electrons. As the electrons collide with the atoms in the sample, they produce various signals which can be detected and measured [18]. These signals provide information about the surface topography and composition of the sample. Microphotographic images from SEM have been provided in Figure 3a,b,c,d.

Among all types of carbon allotropes, only graphene, graphite, and CNTs show electrical conductivity. On the other hand, the carbon films also show conducting behavior. This implies that the grown carbonaceous materials belong to one of the above types of graphitized carbon. With reference to similar images from carbon materials published in the literature [19-21], it can be observed by comparison that the scanned material is composed of carbon.

### Results of optical emission spectroscopy

**Figure 4:**
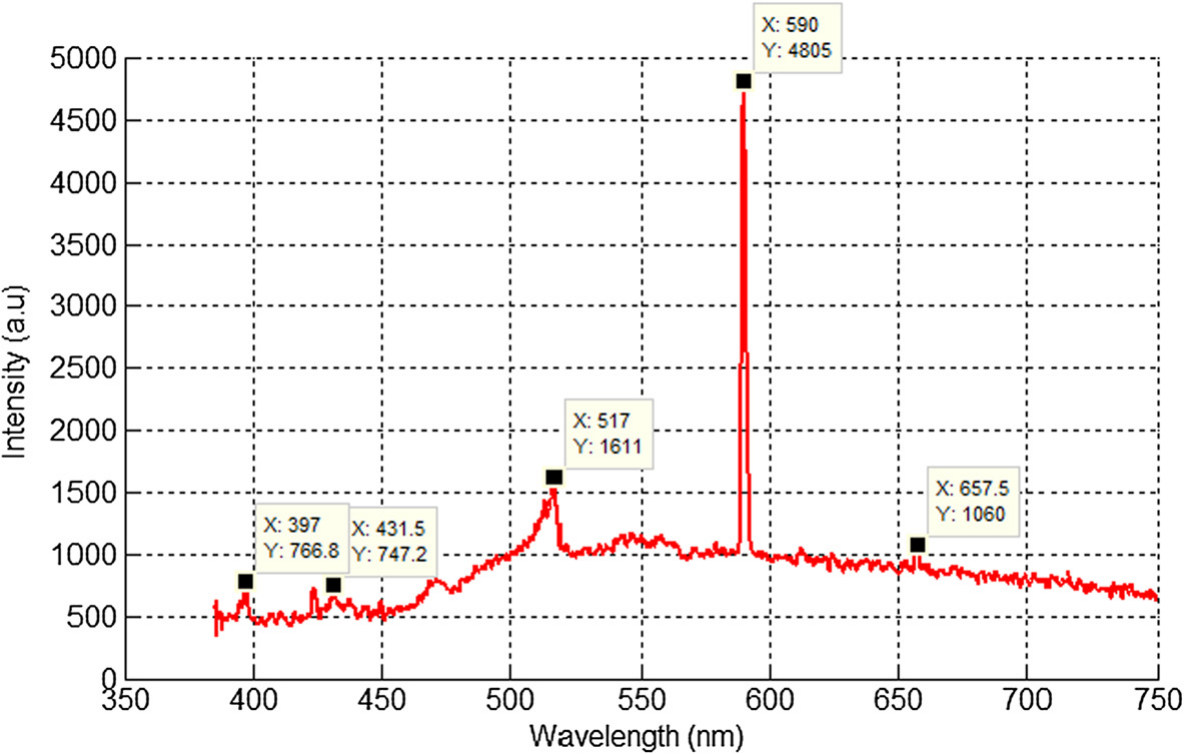
OES spectrum of first phase of evolved species of methane.

**Figure 5:**
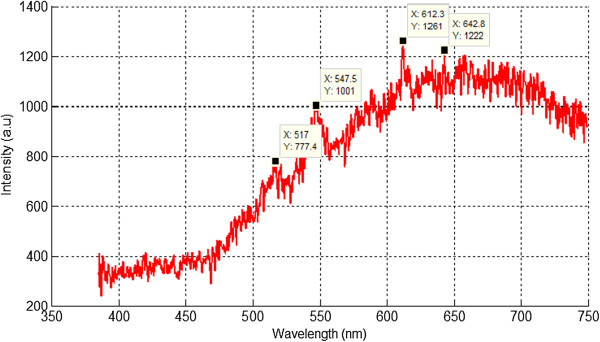
OES spectrum of second phase of evolved species of methane.

The optical emission during arc discharge decomposition was captured in the wavelengths ranging from 385 to 750 nm through a spectrophotometer (StellarNet, Tampa, FL, USA), and the data of the recorded spectra was sketched using MATLAB software. Three evolved peaks of methane species were prominent which belong to CH, C_2_, and H_α_ as shown in Figures 4 and 5. As illustrated in Figure 4, the spectrum consists of the evolved phase of ionized species of methane which indicates peaks of CH at 397 and 431 nm, swan band C_2_ appearing at 516.75, and hydrogen H_α_ appearing at 657.33 nm. These species represent the phenomenon of methane cracking under high-voltage arc discharge conditions as described in recent studies [22]. As soon as the gas breakdown occurs, plasma species will react with each other through ionization and recombination, and the gas enters another phase as shown in Figure 5 which is similar to black body curve. This phenomenon reflects the burning effect of carbon species during carbon deposition on sensor template. It was observed that the carbon agglomeration occurs at high temperatures which helps in the deposition of carbon between the electrodes on the PCB-designed sensor templates [15].

The results of the evolved species in the second phase are different from initial ionization process of pure methane regarding the evolved species. In the second phase, the high peak belongs to C_2_ radical which also indicates that the concentration of C_2_ is much higher in the methane plasma than the other evolved species. The second spectrum also indicates the pyrolysis process of gaseous hydrocarbons that causes carbon deposition between electrodes. The evolved species consist of swan band C_2_ which appears at 516.75 nm and C_2_ at 590 nm, while the two peaks corresponding to hydrogen H_α_ and CH are absent.

The appearances of the peaks in the spectra of both phases of pure methane are listed in Table 2.

**Table 2:** Species of pure methane evolved during decomposition process

**Species**	**Wavelength (nm)**	**Excitation energy (eV)**	**Remarks**	
			**Evolved in first phase**	**Evolved in second phase**
CH	397	-	Yes	No
	431.4	2.9	Yes	No
C_2_	516.75	3.4	Yes	Yes
	590	-	Yes	No
H_α_	657.5	3.3	Yes	No

### Measurements of electrical characteristics

**Figure 6:**
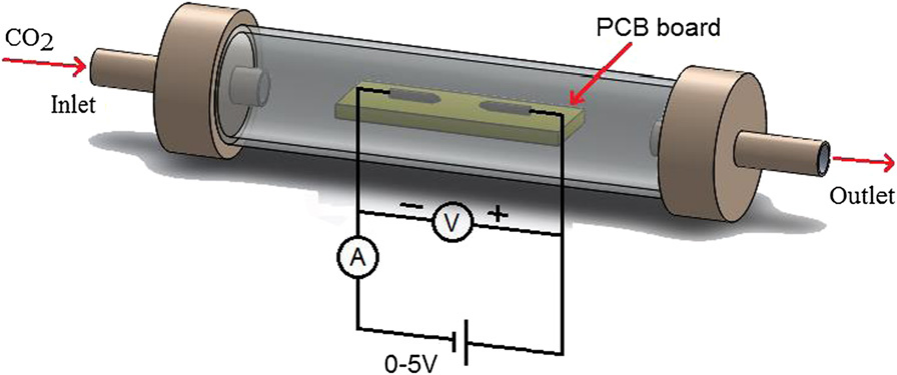
Electrical measurement setup for the carbon film grown between different electrode configurations.

Once the carbon film was produced, a series of low DC voltage measurements were conducted on them in order to reveal their actual current-voltage characteristics. To do this, a DC power supply was employed to apply low voltage to the two electrodes and the carbon film in between. Figure 6 provides a schematic of the electrical circuit implemented in the measurements. The voltage was increased from 0 to 5 V, and the corresponding currents passing through the circuit were recorded using a micro-Ampermeter.

## Results and discussion

**Figure 7:**
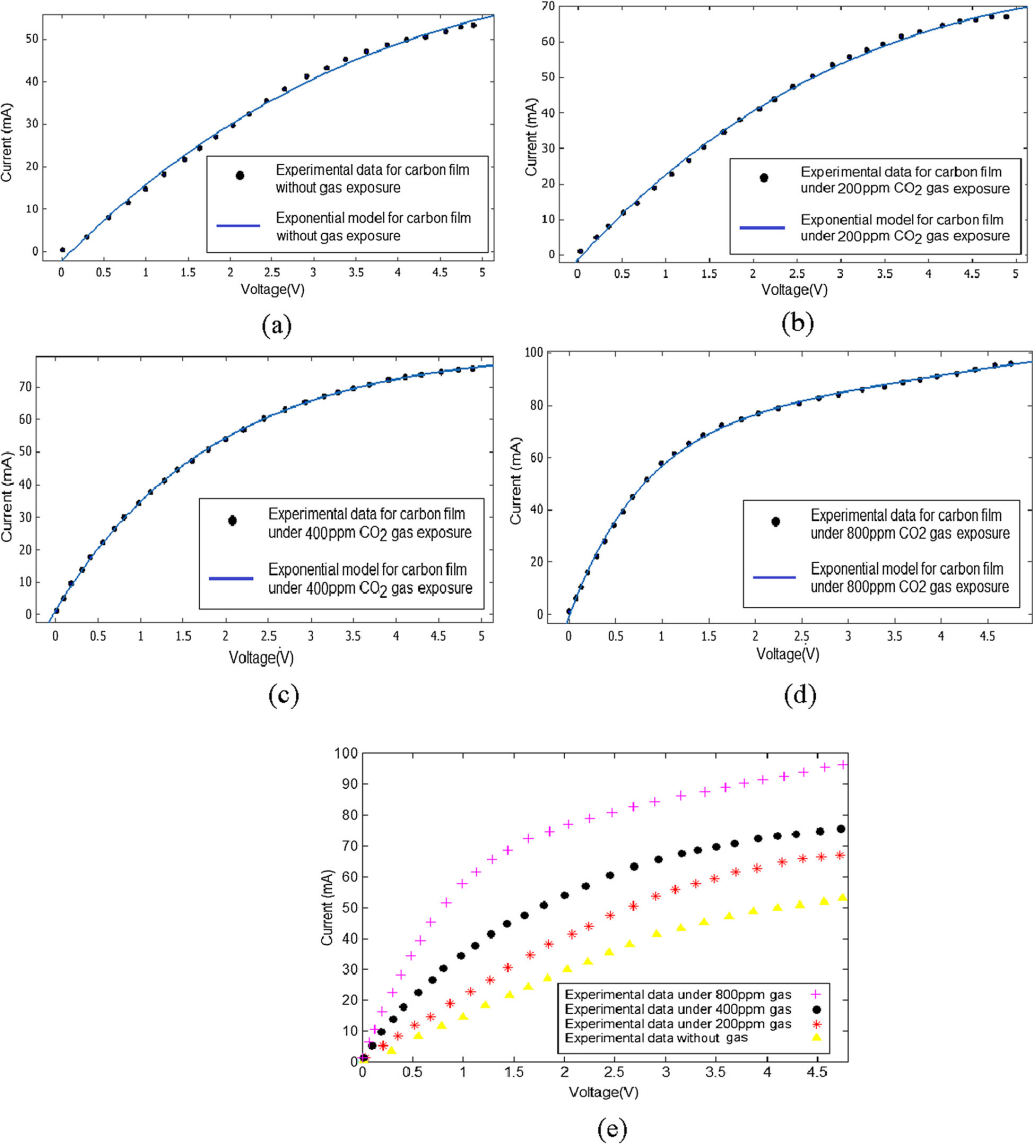
**I-V characteristics of carbon film. (a)** Before gas exposure, **(b)** under 200 ppm gas, **(c)** under 400 ppm gas, **(d)** under 800 ppm gas, **(e)** all experimental tests.

After growing the carbon film, both sides of the chamber must be opened to release the methane gas inside it. After about 20 min, when there is almost no gas present in the chamber, we start to apply the DC voltage and measure the resulting I-V characteristics. The measurements were repeated in the presence of gas with concentrations of 200, 400, and 800 ppm. The current-voltage readings are provided in Figure 7a,b,c,d,e.

In the presence of gas, higher values of current were read which proves the higher conductivity when the carbon films are subjected to gas. Also, as the concentration of gas was increased from 200 to 800 ppm, the current passing through the channel increased further. This phenomenon can be explained by the fact that gas molecules are adsorbed on the carbon film surface and will increase channel conductivity.

In the next step of the study, in order to provide a platform for analytical investigations, MATLAB software was used to fit a curve of exponential form to the corresponding set of experimental data with maximum accuracy (regressions very close to 1). The resulting formula is in the form of Equation 1.

(1) $$F\left(x\right)=\mathrm{aexp}\left(\mathrm{bx}\right)+\mathrm{cexp}\left(\mathrm{dx}\right)$$

Constants *a*, *b*, *c*, and *d* in Equation 1 and the corresponding regression values as well as *R*^2^, SSE, and RMSE errors are provided in Table 3.

**Table 3:** Values for parameters a, b, c, and d and the corresponding regressions

	**Gas exposure**	***a***	***b***	***c***	***d***	***R***^**2**^	**SSE**	**RMSE**
*F*(*x*) = *aexp*(*bx*) + *cexp*(*dx*)	Without gas	7.859e + 5	−0.1246	−7.859e + 5	−0.1246	0.9973	9.849	0.72
	200 ppm	2.999e + 6	−0.1393	−2.999 + 6	−0.1393	0.9984	18.45	0.9157
	400 ppm	86.1	−0.00067	−92.34	−0.5538	0.9998	2.55	0.3194
	800 ppm	74.04	0.05285	−96. 8	−1.299	0.9988	28.3	1.043

## Conclusion

A set of experiments were carried out to fabricate carbon films using high-voltage arc discharge methane decomposition method. High-resolution optical microscopy as well as OES and SEM imaging techniques were implemented to verify the fact that the substances obtained are carbonaceous materials. The carbon films were then used as the channel in an electrical circuit to measure their current-voltage characteristics. Among all types of carbon allotropes, only graphene, graphite, and CNTs show electrical conductivity. On the other hand, the carbon films also show conducting behavior. This implies that the grown carbon films belong to one of the above types of graphitized carbon. It was observed that higher currents pass through the channel when it is exposed to higher concentrations of gas. A mathematical model was obtained for the experimental results using MATLAB curve fitting tool. With the aid of this mathematical representation, it will be possible to characterize and predict the electrical behavior of the carbon films. This will provide a reliable mathematical model which can be used in gas sensing applications to minimize the need for conducting experimental studies.

## Competing interests

The authors declare that they have no competing interests.

## Authors’ contributions

EA carried out the experimental study as well as data collection and analysis, and drafted the manuscript. AE contributed in performing the experiment and also checked the language coherence and technical accuracy of the manuscript. MTA provided the fundamental knowledge and supervised the process and procedure of the experimental study. He also checked for technical and scientific errors.AN applied some optimizing modifications in the programming of the simulation study and also collaborated in the final proofreading. All authors read and approved the final manuscript.
